# Editorial: Psychological studies in the teaching, learning and assessment of mathematics

**DOI:** 10.3389/fpsyg.2024.1389267

**Published:** 2024-03-18

**Authors:** Yiming Cao, Shuhua An, Zsolt Lavicza, Lianchun Dong

**Affiliations:** ^1^School of Mathematical Sciences, Beijing Normal University, Beijing, China; ^2^College of Education, California State University Long Beach, Long Beach, CA, United States; ^3^School of Education (STEM Education), Johannes Kepler University, Linz, Austria; ^4^College of Science, Minzu University of China, Beijing, China

**Keywords:** mathematics, teaching, learning, assessment, psychological studies

## 1 Mathematics teachers and teaching

### 1.1 Mathematics teachers' knowledge and emotion

Jiang, Zhang, Jiang et al. compare how pre-service and in-service mathematics teachers understand mathematics problem solving and its teaching. They reported that pre-service mathematics teachers with postgraduate degrees did not understand mathematics problem solving significantly different from in-service mathematics teachers. They discussed the impacts of postgraduate education, teaching experiences and in-service professional development on the knowledge of mathematics problem solving as well as its teaching.

Wang L. et al. use structural equation modeling to examine how organizational support and job crafting contribute to the occupational wellbeing of the new mathematics teachers in primary and junior high schools. They found that organizational support, job crafting and basic psychological needs have prominent and positive effects on the occupational wellbeing. In addition, there exists a mediating effect of basic psychological needs with organizational support, job crafting, and the teacher wellbeing.

Wang G. et al. investigate secondary school mathematics teachers' autonomous learning capacity from four sub-dimensions: (1) development of study plans, (2) proficiency in using learning methods, (3) learning habit formation, and (4) evaluation of learning outcomes. They reported that secondary school mathematics teachers' overall autonomous learning capacity or the sub-dimensions vary significantly according to teachers' gender, age, years of teaching experience, educational stage, and location (i.e., rural or township). However, teachers with higher qualifications (e.g., doctoral degrees) and job titles (e.g., senior teachers) did not show better autonomous learning capacity than other teachers.

### 1.2 Mathematics instructional practices

Sim and Mohd Matore examine primary school mathematics teachers' usages of five teaching styles, i.e., personal model teaching style, expert teaching style, formal authority teaching style, delegator teaching style, and facilitator teaching style. They found that mathematics teachers' adoptions of teaching styles are weakly correlated with their teaching experiences, implying that teaching experience in mathematics might influence the adoption of teaching styles.

Jiang, Zhang, Ruan et al. conduct a comparative analysis of the extent to which high school mathematics teachers' audible teaching language was satisfied by students. They found that there were no significant differences regarding students' overall satisfaction on expert, skilled, and novice mathematics teachers' audible teaching language. However, in terms of the tone and adaptability of the audible teaching language, students were reported to be more satisfied with expert mathematics teachers than with novice teachers.

Ayabe et al. design problem-appropriate diagram instruction by integrating appropriate line diagrams, tables, and graphs into high school mathematics problem-solving instruction, and examine the effects on students' ability in mathematics word problem solving. They find that the inclusion of problem-appropriate diagrams helps to improve students' problem solving performance and to reduce perceived cognitive load in problem solving activities. The results highlight the importance of selecting relevant diagrams according to the problem-solving tasks to enhance the quality of mathematics problem solving instruction.

Zhao W. et al. conduct a video analysis of dialogue types in expert and novice mathematics teachers' lessons and presents the characteristics of effective mathematics classroom dialogues. They report some culturally unique aspects of effective mathematics classroom dialogues in Chinese contexts, such as high proportion of discourse about basic knowledge, justification, and probing. The contributes to a better understanding of effective classroom dialogues by including practices and perspectives in Chinese contexts.

## 2 Processes of mathematics learning

### 2.1 Origins of affective factors in mathematics learning

Cheng et al. investigate the factors that have impacts on university students' learning satisfaction in the blended learning mode. They find that factors in learning dimension (e.g., learning interest, learning awareness, learning self-efficacy, learning concentration, learning reflection, and the level of communication and interaction) have primary impacts on students' satisfaction with blended learning. It highlights the significance of developing students' learning attitudes in blended learning mode.

Reschke et al. make attempts to explain the gender differences in the early development of students' math self-concepts by examining teachers' judgments of students' mathematics ability and students' perception of teachers' judgments. They find that students' self-concepts can be predicted by both teachers' judgments of students' mathematics ability and students' perception of teachers' judgments. They also find the mediation effect of teachers' judgments on students' self-concepts through students' perceived teachers' judgments. They argue that primary school students tend to have a math-male stereotype when internalizing their teachers' judgments of mathematics ability, leading to girls' lower math self-concepts.

Yesuf et al. examine what factors contribute to high school students' mathematics self-efficacy. They report that living arrangements with parents (i.e., living with single parent or both parents), students' expected grade in the upcoming national exam and expected marks in the semester can significantly predict students' mathematics self-efficacy, whereas students' received tutorial, students' plan for further education and professional aspiration do not significantly influence students' mathematics self-efficacy.

### 2.2 Relations between affective factors and mathematics learning

Xu et al. use bibliometric analysis techniques to review the existing studies regarding growth mindset and mathematics learning. They find that there is a lack of studies focusing on student's mindset in the specific sub-fields of mathematics, e.g., algebra, calculus, or geometry. In addition, few studies addressed how teachers' mindset and parents' mindset make impacts on students' mathematics learning. The findings provide implications for future explorations about growth mindset and mathematics learning.

Dong et al. examine the functioning processes of growth mindset in mathematics learning by combining students' growth mindsets, failure attributions, intrinsic motivation, mathematics self-efficacy, mathematics anxiety and mathematics achievements in one statistics model. They find that students' growth mindset doesn't directly predict their mathematics achievements, but indirectly influences mathematics achievements through students' intrinsic motivation. Failure attributions, mathematics self-efficacy, and mathematics anxiety play sequentially mediating roles in the relation between students' growth mindset and their academic achievements.

Sakellariou examines the reciprocal relationship between students' academic self-efficacy and mathematics achievements in high school. It is reported that there are robust reciprocal effects between self-efficacy and mathematics achievement for boys, and the dominant effect is from earlier achievement to later self-efficacy. However, there are no strong evidences for such reciprocal effects in the sample of girls. This study supports the existence of higher reciprocal effects for boys than girls, providing evidences for significant gender differences in the context of high school mathematics learning.

Zuo and Wang explore the relationship between mindfulness-based intervention (MBI) and high-risk students' mathematics achievements in middle school by considering the mediating roles of mathematics-specific exam anxiety and mathematics self-efficacy. The results show that mindfulness can significantly improve students' mathematics achievements, and reduce students' mathematics anxiety, as well as problem-solving obstacles resulted from mathematics anxiety. This study provides evidences for mindfulness intervention's efficacy in promoting middle school students' mathematics academic performance.

Brumm and Rathgeb-Schnierer explore the relationship among accuracy in numerosity estimation, mathematics achievement, and mathematics interest in the sample of primary school students. The results indicate that there is no statistically significant association either between accuracy in numerosity estimation and mathematics interest or between accuracy in numerosity estimation and mathematics achievement. This study suggests the needs for further studies regarding the function of numerosity estimation in mathematics learning.

Guo et al. investigate the relations between primary school students' filial piety beliefs and mathematics procrastination by taking into account the mediating role of academic emotions (e.g., enjoyment and anxiety). The results show that, students with reciprocal filial piety tended to have fewer procrastination behaviors in mathematics learning than students endorsing authoritarian filial piety. Reciprocal filial piety leads to more enjoyment in mathematics learning, which may in turn establish a protective mechanism against procrastination. This study contributes to a better understanding of the roles of students' filial piety beliefs in mathematics learning.

### 2.3 Environmental factors in mathematics learning

Yu et al. compare how primary and middle school students' relationships with parents, teachers, and their peers make impacts on their academic performance in mathematics. They report that, compared with students' relationships with parents, teachers, the quality of peer relationships was more closely associated with academic achievement in mathematics. The results highlight the significant roles of peer relationships in mathematics learning.

### 2.4 Cognitive processes of mathematics learning

Zhao J. et al. examine students' conflict discourse in mathematics cooperative problem solving, analyzing the discourse style and language characteristics of the three stages of conflict discourse (i.e., Initial stage of conflict, Conflict and Negotiation stage, and the end of conflict stage). They identify twelve categories of conflict discourse, count each categories' frequencies, and examine the processes of how conflicts in mathematics cooperative problem solving can be intensified or resolved by different categories of conflict discourses.

Shang et al. use eye-tracking techniques to examine how structured stepwise presentations make impacts on primary school students' attention in mathematics learning and their learning outcomes in fraction. The results suggest that structured stepwise presentation can better orient student to pay attention to connecting relative elements in fraction learning and contribute to more desirable learning performance in fraction. This study supports the importance of designing structured stepwise presentations in mathematics teaching.

Wan Hussin and Matore explore the impacts of secondary school students' learning styles in mathematics (i.e., visual, auditory, and kinesthetic) on their academic procrastination in mathematics. They find that visual learning styles significantly contribute to academic procrastination in mathematics. Visual learning style requires long-term memory and strong visual skills, which may cause pressure and depression in mathematics learning and in turn lead to academic procrastination in mathematics. In addition, academic procrastination in mathematics decreased for students with kinesthetic learning style and this might be attributed to kinesthetic students' preferences to using hands-on or applications with real situations, which promote their interest and motivation in learning mathematics. This study suggests the necessity of considering students' learning styles when coping with procrastination in mathematics learning.

Jiang and Li examine how secondary school students use mathematics textbooks in mathematics learning. They report that, secondary school students used mathematics textbooks for various reasons, such as developing mathematical knowledge, skills, and abilities, but they were reluctant to believe the connection between mathematics textbooks and their mathematics exam scores. In addition, secondary school students use mathematics textbooks significantly according to their school regions, grade levels, and teachers' profiles. This study contribute to a better understanding of mathematics textbooks by considering students' perspectives.

### 2.5 Cultivation of high-order thinking skills

Wang T. et al. examine the how middle school students' mathematical modeling competency influence creativity. The results confirm the significant association between mathematical modeling competency and creativity, as well as the mediating roles of curiosity in the association. This study highlights the effects of mathematical modeling competency on creativity, implying the possible way of developing middle school students creativity via the improvement of their mathematical modeling competency.

Ji and Guo conduct a meta-analysis of the relationship between working memory and mathematical problem solving. The results show that, students' ability to solve dressed-up word problems are more strongly correlated with working memory than their ability to cope with intra-mathematical problems. In addition, compared with other components of working memory, the central executive function is more strongly associated with mathematical problem solving ability. Gender ratio shows significant moderating effects and the association is stronger in the samples of boys than in the sample of girls. This study helps to clarify the roles of working memory in mathematical problem solving rather than in the general mathematics learning.

Shi et al. use the functional near-infrared spectroscopy (fNIRS) technique to investigate the effects of middle school students' hands-on experience on geometry learning by considering academic level as an important impacting factor. The results show that, hands-on operation with concrete geometric manipulatives, in contrast to observation, can better enhance the activation of sensorimotor systems, which are important in geometry representation and processing and in turn contribute to better geometry problem-solving performance. This study helps to uncover the mechanism in which hand-on experience promote middle school geometry learning.

## 3 Assessment in mathematics education

### 3.1 Assessing contributing factors in mathematics learning

Piccirilli et al. assess the influence of mathematics anxiety on vocational secondary school students' calculus learning. They argue that, assessing secondary school students' level of mathematics anxiety at the beginning of the school year can help to identify the students who have the potential risk of failing in the subsequent calculus learning. The study suggests the consideration of mathematics anxiety assessment as a tool to promote secondary school students' mathematics learning.

Lin and Chen present the processes of develop the students' mathematics self-directed learning scale containing four sub-scales with 50 items and apply the scale to measure high school students' mathematics self-directed learning. The results show the existence of gender differences in mathematics self-directed learning and male students scored higher in mathematics self-directed learning than female students. However, students' mathematics self-directed learning does not increase with the grade level in high school students. This study provides a reliable and valid tool to assess students' self-directed learning in mathematics and helps to include the development of mathematics self-directed learning in mathematics curriculum reform.

Uesaka et al. use item response theory to analyze university students' acquisition and usages of different types of learning strategies, showing that the average levels of students' strategy acquisition are associated with their academic achievement ranking in university learning. This study proposes a framework for assessing university students' learning strategies' levels and suggests to provide university students with learning resources and advice according to their level of acquisition of learning strategies.

### 3.2 Assessing mathematics competencies

Zhang et al. utilize DINA (Deterministic Inputs, Noisy, and Gate) model to diagnose fourth grade students' mathematical ability in calculation by presenting each student's arithmetic knowledge status. They report students' mastery of ten cognitive attributes in mathematics ability and identify various cognitive error patterns and major cognitive error patterns in arithmetic. This study provides researchers and practitioners with the tool for students' diagnosis in mathematics learning, which is helpful for designing targeted remediation programs in primary mathematics teaching.

Wu et al. employ cognitive diagnostic assessment (CDA) to construct a cognitive model for middle school students' data analysis ability, allowing to accurately detect students' knowledge structure or operational skills in data analysis, and to present students' data analysis ability's learning path and learning progression. This study contributes to the inclusion of a new cognitive diagnostic perspective on the assessment of middle school students' data analysis abilities.

Meng et al. employ bi-factor theory and propose a full-information item bifactor (FIBF) model to measure primary and middle school students' mathematical ability. They also apply the FIBF model in a large-scale data set to examine the performance of the model. The results show that FIBF model is better fitting than other models such as UIRT and MIRT models, and a more reasonable interpretation can be obtained by using the ability scores from the FIBF model. This study supports the feasibility of employing the FIBF model in large-scale mathematics testing projects.

## Author contributions

YC: Writing—review & editing. SA: Writing—review & editing. ZL: Writing—review & editing. LD: Writing—original draft.

